# Identification of plastic-associated species in the Mediterranean Sea using DNA metabarcoding with Nanopore MinION

**DOI:** 10.1038/s41598-020-74180-z

**Published:** 2020-10-16

**Authors:** Keren Davidov, Evgenia Iankelevich-Kounio, Iryna Yakovenko, Yuri Koucherov, Maxim Rubin-Blum, Matan Oren

**Affiliations:** 1 Department of Molecular Biology, Ariel University, Science Park, 40700 Ariel, Israel; 2 Israel Oceanographic and Limnological Research, National Institute of Oceanography, Tel Shikmona, 31080 Haifa, Israel

**Keywords:** DNA, Bioinformatics, Sequencing, Next-generation sequencing, Microscopy, Scanning electron microscopy, Biodiversity, Ecosystem ecology, Microbial communities, Environmental microbiology, Biological techniques, Ecology, Microbiology, Ecology, Biodiversity, Environmental sciences, Environmental impact, Ocean sciences, Marine biology

## Abstract

Plastic debris in the ocean form a new ecosystem, termed ‘plastisphere’, which hosts a variety of marine organisms. Recent studies implemented DNA metabarcoding to characterize the taxonomic composition of the plastisphere in different areas of the world. In this study, we used a modified metabarcoding approach which was based on longer barcode sequences for the characterization of the plastisphere biota. We compared the microbiome of polyethylene food bags after 1 month at sea to the free-living biome in two proximal but environmentally different locations on the Mediterranean coast of Israel. We targeted the full 1.5 kb-long 16S rRNA gene for bacteria and 0.4–0.8 kb-long regions within the 18S rRNA, ITS, tufA and COI loci for eukaryotes. The taxonomic barcodes were sequenced using Oxford Nanopore Technology with multiplexing on a single MinION flow cell. We identified between 1249 and 2141 species in each of the plastic samples, of which 61 species (34 bacteria and 27 eukaryotes) were categorized as plastic-specific, including species that belong to known hydrocarbon-degrading genera. In addition to a large prokaryotes repertoire, our results, supported by scanning electron microscopy, depict a surprisingly high biodiversity of eukaryotes within the plastisphere with a dominant presence of diatoms as well as other protists, algae and fungi.

## Introduction

Plastic pollution has become an integral part of our environment. Circulating since the 1950s, plastic traces are now present almost everywhere on earth^1^. In recent years, an order of magnitude estimate of 10 million tons per year of plastic litter finds its way to the oceans^2,3^. Marine plastic debris is generally highly persistent and remain in the environment for a long time^4^, serving as stable substrates for the colonization and growth of a variety of marine organisms and microorganism communities^5^. The plastic ecosystem is distinct in its biota composition from that of its surrounding water^6,7^ and consequently was termed “plastisphere”^6^.

The development boost in next-generation sequencing (NGS) platforms in recent years, significantly reduced the time and costs of sequencing, transforming the environmental metabarcoding and biodiversity research^8^. Following pioneer studies, such as those of De-Tender et al.^9^ and Zettler et al.^6^, researchers have begun using NGS-based metabarcoding as the major tool to depict the plastisphere taxa composition on marine microplastic collected from the ocean and on plastic polymers that were experimentally exposed to the marine environment. In most of these studies, short regions of the small ribosomal subunit ribonucleic acid 16S gene (16S rRNA) and/or of the equivalent eukaryote 18S rRNA gene have been used for metabarcoding with second-generation sequencing technologies, predominantly MiSeq Illumina sequencing as well as 454 pyrosequencing (Roche)^10^.

Whereas this approach has yielded important data about the plastisphere composition, it is yet limited in its resolution and taxonomic coverage. Indeed, second-generation sequencing technologies have little error rates and excellent coverage, which is advantageous for environmental metabarcoding. However, these technologies produce short reads, forcing researchers to choose short barcoding regions, typically within 200–500 bp length range^11^. The short barcode sequence length limits the barcoding resolution, which often fails to discriminate among taxa due to the ambiguity in the read taxonomic classification^10^. Hence, using long-read sequencing technologies, such as third-generation Pacific Biosystems SMRT and Oxford Nanopore sequencing, to sequence longer regions of the same barcode loci is expected to result in better classification of taxa^10,12,13^. Moreover, current plastisphere metabarcoding studies are usually based on one or two barcode loci, an approach which limits taxonomic identification capacity. This is especially true for eukaryotic microorganisms that are extremely heterogeneous^14^. Different variable regions of the 18S rRNA including V4, V7 and V9 regions were used for the metabarcoding of eukaryotic microorganisms in the plastisphere^11^. Yet, some relevant key taxonomic groups are underrepresented or even missing from the resulting 18S metabarcoding datasets^15,16^. Relying on additional barcode loci may aid in filling this gap^15^. For example, primers for the *tufA* gene, which encodes the elongation factor EF-Tu, were found to perform better in the identification of certain marine microalgae species^17^, the nuclear ribosomal internal transcribed spacer (ITS) was found to be more suitable for the identification of fungi^18^ and the cytochrome *c* oxidase I (COI) is more suitable for covering metazoans^19^. For prokaryotes, the most frequently used genetic barcodes in environmental metabarcoding studies lies within the hypervariable 16S rRNA V3–V5 locus for bacteria^11^ and within V1–V2 locus for archaea^20^. However, as is the case for eukaryotes, their short sequence length (0.3–0.5 kb) may limit their taxonomic resolution^10^.

In this study, we performed metabarcoding of species based on DNA that was extracted from seawater and from polyethylene (PE) plastic bags that were submerged for 1 month in two proximal but environmentally different marina and open-water locations at the Mediterranean coast of Israel. We used the Nanopore MinION sequencing platform to sequence the full 16S rRNA gene for bacteria (V1–V9 region, ~ 1.5 kbp long) and a combination of four established eukaryotic barcodes within the 18S rRNA (~ 0.7 kbp), ITS (~ 0.4 kbp), *tufA* (~ 0.8 kbp) and COI (~ 0.7 kbp) genetic loci. We first tested the barcoding efficiency in the identification of known species using MinION sequencing for each of the five loci. We later used the same barcodes to profile the taxonomic diversity in pooled plastisphere and water samples from each location using a single Nanopore MinION flow cell with multiplexing. Our metabarcoding results, together with the morphological identification of taxa by scanning electron microscopy (SEM), demonstrate the efficiency of this approach in the characterization of life in the plastisphere as well in the identification of plastic-associated species.

## Methods

### Experiment setup

**Figure 1:**
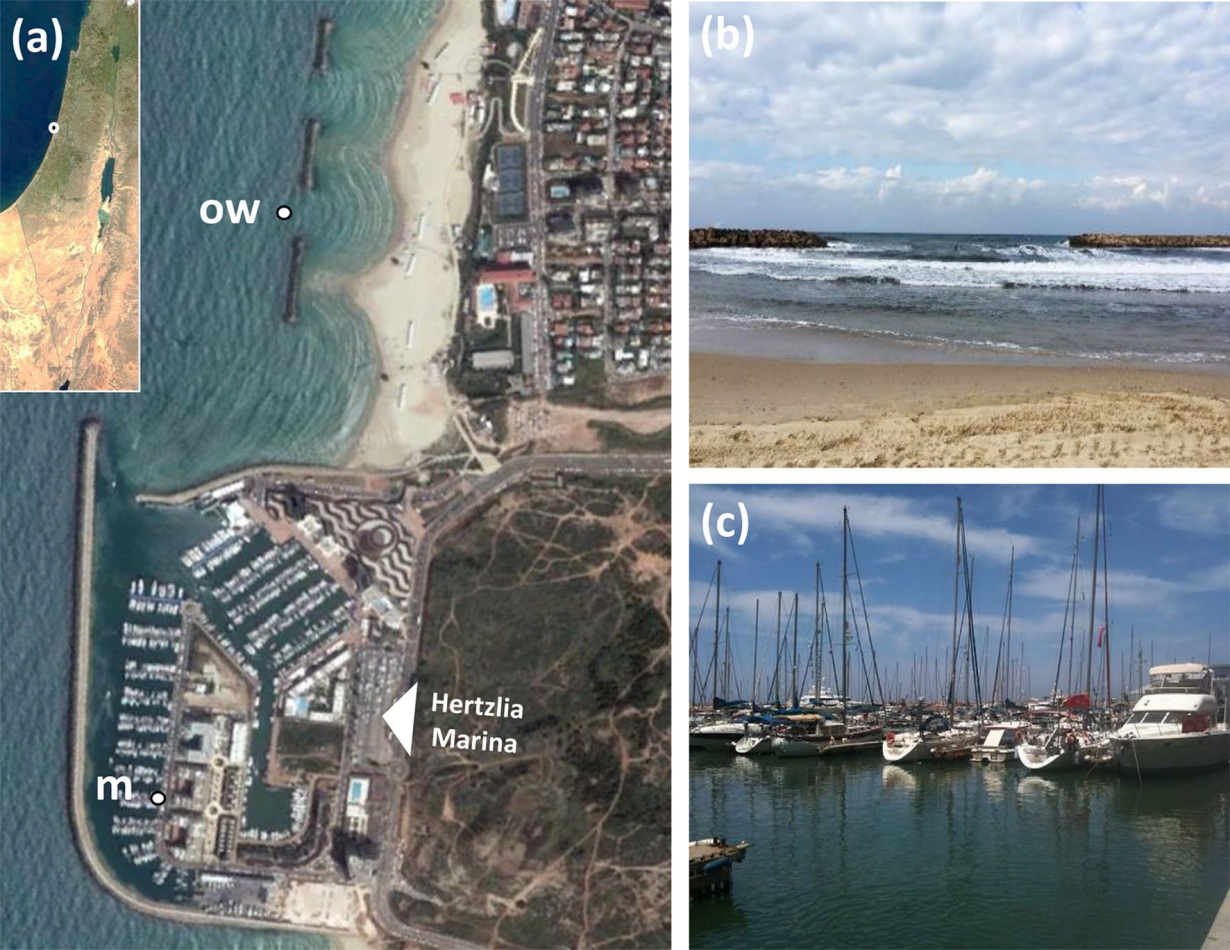
Experiment location. (**a**) Top view. Inset shows location in Israel (**b**) open water location—side view. (**c**) Marina location—side view. *OW* open water location, *m* marina location.

For the characterization of the plastisphere taxa composition on low-density polyethylene (LDPE) we used plastic food bags (18 × 20 cm) that were positioned in Herzliya marina (32° 09′ 38.8" N 34° 47′ 35.0" E) and in the open water of the nearby Herzliya public beach (32° 10′ 05.4" N 34° 47′ 52.5" E, ~ 300 m of the shore) (Fig. 1). The bags were secured to a stable structure (i.e. buoy/metal post/dock cleats) with a fishing line sewed into a firm polycarbonate strip that was inserted in one edge of each of the bags allowing limited movement but preventing tearing and detachment. The bags were positioned 20–30 cm below the surface and ~ 6 m above the sea bed. Bags were submerged for 1 month (December 20, 2018, to January 20, 2019) until sampling.

### Plastic and water sampling

Water and polyethylene (PE) food bag samples were taken from the marina and open water locations. Six pieces of the polyethylene were taken for DNA extraction and SEM imaging from the PE food bags in each location. The pieces (~ 1 × 2 cm) were cut using sterile scissors, gently washed 3 times for 5 min with filtered seawater (FSW) to remove unbound material and separately processed in each of the subsequent assays and procedures described below. Seawater was sampled 3 times in proximity to the PE bags using a sterile plastic sampling bottle. 0.5 L of the sampled water was filtered on 0.22 µm polyethersulfone membrane (Millipore) using a 20 L/min pump (MRC).

### SEM microscopy

The visualization of the microorganisms on the PE was performed by scanning electron microscopy (SEM). PE samples were fixed for 2–5 h in 1% glutaraldehyde and 4% PFA and post-fixed with osmium tetroxides (OsO4) for one hour at room temperature followed by washes (three times for 5 min) in distilled water. Samples were kept in 50% ethanol in phosphate-buffered saline (PBS) at − 20 °C until use. One day before use, samples were dehydrated in graded ethanol series for 10 min each in 50%, 70%, 85%, 95% ethanol, followed by 3 × 15 min in 100% ethanol. Dehydrated samples were air-dried for at least 5 h in a hood, sputter-coated with 10 nm of platinum/gold (Quorum Q150T ES) and then visualized and imaged on an Ultra-High Resolution Maia 3 FE-SEM (Tescan) in a range of 3–7 kV voltage.

### DNA extraction

DNA was extracted using the phenol–chloroform extraction method. The samples (filters and PE) were collected into 15 mL Eppendorf tubes containing 2 mL of lysis buffer (10 mM Tris–HCL pH8, 25 mM Na_2_EDTA pH8, 1v/v% SDS and 100 mM NaCl) and stored in − 20 °C until processing. Samples were later thawed, subjected to bead beating with  ~ 0.4 gr of 425–600 µm sterile glass beads (Sigma) and proteinase K (5 units/µL) and Lysozyme (2000 units/µL) digestion. All other steps of the DNA extraction were performed according to^21^. DNA was finally eluted in 40 µL EB (10 mM TE Tris 1 mM EDTA pH8).

### PCR amplification and cleanup

Each sample was subjected to PCR amplification with five sets of commonly used primers to amplify five barcode regions from the 16S^22^, COI^23^, 18S^24^, *tufA*^25^ and ITS^26^ loci. The choice of barcoding primer-sets and loci (Table 1) was so that they collectively cover a wide range of marine plastic-relevant taxa. The reaction volume was 50 µL with 25–75 ng of template sample DNA. The amplification of barcodes was performed according to the parameters in Table 1. PCR product (3 µL) was tested on 1% agarose gel (50 V, 40 min) alongside the DNA size marker. Cleaning of the PCR products was performed with QIAquick-PCR Purification kit (QIAGEN). The cleaned barcode amplification products were measured with NP-80 spectrophotometer (A_2_S) and Qubit 3 fluorometer (Invitrogen by Thermo Fisher Scientific) to assure sufficient quantity and quality for nanopore sequencing according to oxford nanopore requirements.

**Table 1:** Barcode amplification details.

Gene/genetic locus	Primers	Primer sequences (5′ to 3′)	Annealing temp. (ºC)	Amplicon Length (bp)
16S-small subunit ribosomal RNA	27-F 1492-R	AGAGTTTGATCMTGGCTCAG GGTTACCTTGTTACGACTT	56	1500
18S-small subunit ribosomal RNA	566-F 1289-R	CAGCAGCCGCGGTAATTCC ACTAAGAACGGCCATGCACC	57	723
cytochrome c oxidase subunit I (COI)	LCO1490-F HCO2198-R	GGTCAACAAATCATAAAGATATTGG TAAACTTCAGGGTGACCAAAAAATCA	46	710
*tufA*-elongation factor tu (gene)	tufGF4-F tufA-R	GGNGCNGCNCAAATGGAYGG CCTTCNCGAATMGCRAAWCGC	45	807
ITS-internal transcribed spacer region in the rRNA	ITS86-F ITS4-R	GTGAATCATCGAATCTTTGAA TCCTCCGCTTATTGATATGC	55	369

PCR program: 2 min at 94 °C, 32 cycles of: 30 s at 94 °C, 30 s at 45 °C to 57 °C, 30–60 s at 72 °C, and final extension 72 °C for 5 min.

### MinION library preparation and multiplexed nanopore sequencing

The sequencing libraries were prepared using the 1D Native barcoding genomic DNA protocol with EXP-NBD104 and SQK-LSK109 kits (Oxford Nanopore Technologies). ~ 200 fmol of purified amplification products were subjected to DNA repair and end-prep using a NEBNext DNA repair mix and NEBNext Ultra II End Repair/dA-Tailing Module (New England Biolabs). The library preparation included two ligation steps. In the first step, multiplexing barcodes were ligated to 500 ng of each of the processed amplification products, using T4 Ligase (New England Biolabs). Equal molarities of the barcoded amplicons were pooled together and 700 ng of the pooled sample was subjected to MinION adaptor ligation according to the protocol. Each step was followed by DNA purification with SPRI magnetic beads (Canvax, Spain). For optimal utilization of the MinION flow cell capacity, we multiplexed and sequenced the barcode samples in two separated batches: the first included the 16S rRNA, the 18S rRNA and the COI libraries for each of the four samples (12 multiplexed libraries in total), and the second included the ITS and the *tufA* libraries for each of the four samples (8 multiplexed libraries in total). The sequencing runs were performed on the same MinION cell with a washing step between them according to Oxford Nanopore Technologies (ONT) instructions. The libraries were loaded to the Nanopore MinION Spot-on flow cell (FLO-MIN106D, version R9) and sequenced until reaching ~ 2 Gb (~ 1.8 M reads) for the first batch (figure S1a) and ~ 0.7 Gb (~ 1.1 M reads) for the second batch (figure S1b). Base-calling was done automatically by the MinKnow program. Raw reads were obtained in FAST5 and FASTQ formats from which “pass” quality reads were subjected to further analysis.

### Data analysis and statistics

Following base-calling, reads were demultiplexed and adaptors were trimmed with qcat (https://github.com/nanoporetech/qcat). Primers were removed using Cutadapt using default parameters with 10% error tolerance^27^. Read quality was estimated and visualized using NanoPlot^28^. Sequences were filtered based on read quality (-min_qual_mean 10) and read length was restricted (-min_len X -max_len Y as follows: 16S, 1200–1400 bp; 18S, 650–720 bp; COI, 630–700 bp; ITS, 270–340; *tufA*, 700–1000), using prinseq-lite V0.20.4^29^. Because generating consensus sequences is essential for increasing the accuracy in Nanopore MinION studies^30,31^, we generated consensus sequences for each of the amplicon read datasets using ONTrack pipeline (https://github.com/MaestSi/ONTrack) according to Maestri et al.^32^. The consensus sequences were also used as reference sequences for the calculation of the mapping and error rates using ONTrack Calculate_mapping_rate.sh and Calculate_error_rate.sh scripts^32^. For diversity analyses, trimmed sequences were imported into Qiime2^33^ using the SingleEndFstqManifestPhred33 input format. The reads were dereplicated and clustered into operational taxonomic units (OTUs) at 80% identity (as implemented in^34^). We note that clustering had little effect on the mapping of species, and was implemented to reduce complexity and computation time when performing BLAST searches against large databases such as Silva132^33^. Representative sequences were classified using BLAST with Qiime2 (classify-consensus-blast), using -p-perc-identity 0.8 and -p-maxaccepts 1 against the following databases: for the 16S rRNA gene, we used either the RefSeq^35^ or Silva132 database (the latter was also used for classification of the 18S rRNA gene sequences). ITS sequences were classified against the UNITE database V8 with the dynamic clustering thresholds^35^. Previously curated databases were used to classify *tufA*^36^ and COI^37^ sequences, following manual formatting to fit Qiime2 import. To estimate the taxa diversity among the samples, a PCoA chart was produced with the Bray–Curtis distance matrix based on the operational taxonomic units (OTUs) identities. PCoA plots and heatmaps were produced using Phyloseq^38^ and Ampvis2^39^ packages in R version 3.6.3^40^, based on feature and OTU tables that were exported from Qiime2. Venn diagrams were created with InteractiVenn free platform (https://www.interactivenn.net), based on OTUs that corresponded to at least two reads, to filter out possible contaminations and false matches.

### Nanopore sequences deposit in GeneBank

All Nanopore MinION filtered reads analyzed in this project were deposited in the NCBI SRA database (https://www.ncbi.nlm.nih.gov/sra). The data of the barcodes test run were deposited under Bioproject PRJNA627087 (samples accession numbers: SRR11581740–SRR11581744). The data of the plastic and water samples was deposited under Bioproject PRJNA625720 (samples accession numbers: SRR11554946–SRR11554965).

## Results

### Testing the Nanopore MinION barcoding accuracy and efficiency for the chosen loci

To test the accuracy and the efficiency of the primers and the barcoding pipeline that was followed, we amplified the relevant barcode regions from the genomic DNA of five representative species and sequenced them with MinION using ligation protocol with multiplexing as detailed in the methods section. The selected species were: *Escherichia coli* (16S rRNA gene), *Homo sapiens* (COI), *Gracilaria cearensis* (18S rRNA gene), *Ulva fasciata* (*tufA*) and *Saccharomyces cerevisiae* (ITS), representing bacteria, metazoans, green and red algae and fungi, respectively (Table 2). The mapping rates of the reads to the consensus sequences generated for the five species varied between 85% (*Ulva* tissue) to 100% (*E. coli* cell culture) and was correlated with sample purity level and the presence of foreign DNA contaminants (i.e. unsterile organisms vs. pure cell cultures). The average error rates of individual reads compared to the barcode consensus sequences ranged between 7.9% and 12.18%. However, we succeeded to decrease the error rates to 6.6–10% by filtering the reads according to their base-caller assigned quality (-min_qual_mean 10) (Table 2) and subsequently used this threshold for our experiment. In spite of the high error rate in individual sequences, almost all of the errors may be corrected since they tend to be introduced at random positions and therefore filtered out in the process of consensus sequences generation. Consequently, consensus sequences that will be based on a higher number of reads (up to a limit), are expected to be more accurate. To simulate the effect of MinION read coverage on the accuracy and efficiency of the metabarcoding we re-obtained the different parameters using 400, 100 and 10 reads that were randomly picked from each of the mapped barcode read pools. Overall, the parameters were similar in all runs, with only a slight decrease in the percent identity of the consensus sequence to the database sequence toward 10 reads coverage which was still sufficient for the correct identification of the species. This suggests that using the MinION sequencing platform in the barcoding of species within an environmental sample, may be accurate given these minimal coverage requirements.

**Table 2:** MinION barcoding test run.

Genetic barcode	Target species	Top consensus GeneBank hit accession	Number of reads assigned to barcode	Average error rate for raw reads (%)	Average error rate for filtered reads (%)^a^	Consensus match to target sequence (%)		
						X 400 coverage	X 100 coverage	X 10 coverage
16S	*Escherichia coli*	CP027701.1	11,107	12.18	10	100	99.9	98.4
ITS	*Saccharomyces cerevisiae*	CP022977.1	6001	10	9	100	99.7	98.1
COI	*Homo sapiens*	MG660736.1	6748	10.29	9.4	100	100	99.4
18S	*Gracilaria Ceara*	AF468890.1	22,470	9.86	8.3	100	99.9	99.1
tufA	*Ulva fasciata*	NC_042255.1	6951	7.9	6.6	100	99.9	98.8

^a^Sequences were filtered based on read quality (-min_qual_mean 10).

### The polyethylene prokaryotic community

**Figure 2:**
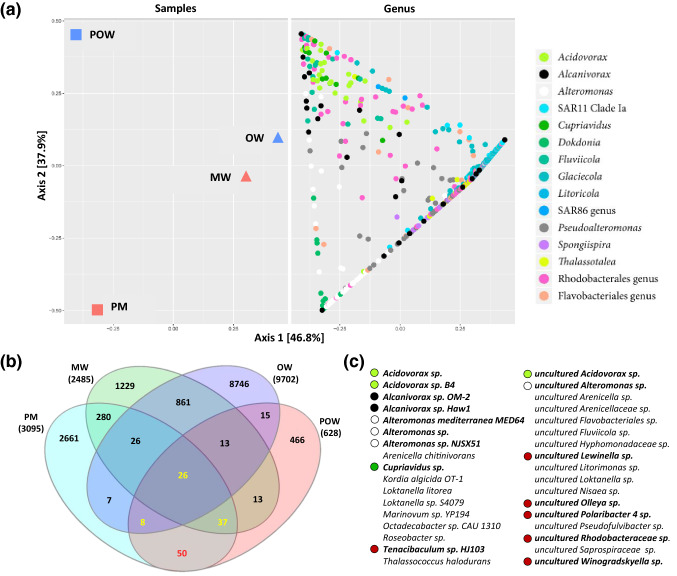
Bacterial genera diversity and PE-specific species. (**a**) Beta-diversity of the bacterial genera and their distribution among the pooled samples (**b**) Shared and unique OTUs among the samples. In yellow—OTUs that were identified in both PE samples, but also in water samples. OTU counts refer to OTUs that corresponded to ≥ 2 reads. In red—OTUs that were unique to the PE samples. (**c**) The list of PE-specific bacterial taxa. Known petroleum and plastic-degrading genera are marked [according to their color in (**a**)]. *OW* open water samples, *POW* open water plastic samples, *MW* marina water samples, *PM* marina plastic samples.

Our MinION-based 16S rRNA metabarcoding pipeline identified 1823 species in the pooled marina water sample (MW), 2572 in the pooled open water sample (OW), 1810 in the pooled marina PE sample (PM) and 1004 in the pooled open water PE sample (POW) with average mapping rate (percent of mapped reads from total reads) of 45% (Table S1). At the phylum level, Proteobacteria were the most dominant in all samples followed by Bacteroidetes and Cyanobacteria. The PE samples contained higher ratios of Bacteroidetes (PM-25% and POW-22%) compared to the water samples (MW-13% and OW-8%) as well as higher ratios of Cyanobacteria (14% and 5% in the PE samples vs. 2% in both water samples) (Fig. S2). These results agree with previous studies that likewise showed higher ratios of Bacteroidetes and Cyanobacteria on plastic compared to the water column^6,7,11^. The analysis of the diversity among the samples (Beta-diversity) suggests that the free-living prokaryote taxa from the two pooled water samples cluster together and are separated from the plastic-attached taxa identified in the pooled PE samples (Fig. 2a). Accordingly, only 0.6% of the OTUs in the open water samples and 6.6% of the OTUs in the marina samples were shared between the PE and the water column (Fig. 2b). These results align with other studies showing that the plastisphere is a separate environmental niche for bacteria^6, 7^.

In the search for plastic-specific bacteria we focused on OTUs that were identified in both PE samples, assuming that the occurrence of these species on PE from two very different environments (marina and open water) implies their strong association to this niche. Based on the 16S rRNA metabarcoding, 121 bacterial OTUs of two reads and above, were identified in both PE samples (Fig. 2b). Within this list, 50 OTUs (0.3% of the total OTU count), corresponding to 34 bacteria species, were identified in both PE samples and not in the water column (Fig. 2c). Almost half of the plastic-specific species belong to genera that are known to be directly involved in plastic or petroleum degradation including *Alcanivorax*^41^, *Acidovorax*^42^ and *Alteromonas*^43^ (Fig. 2c). Additional bacteria of these genera were also enriched in one or both of the PE samples according to taxa diversity analysis among samples (Fig. 2a) and species abundances table (Fig. S3). An extended screen for plastic-associated species (Table S2), revealed taxa that were identified in both PE samples but were also dominant in the marina water sample. At the top of the list in this category was *Pseudoalteromonas* sp. 12 (mapped reads of total reads: PM—0.55%, POW—0.07%, MW—1.95%). Although there is currently no evidence for the ability of this species to degrade petroleum, other *Pseudoalteromonas* species are known as hydrocarbon degraders^44,45^. Another known hydrocarbon-degrading bacterium, *Alcanivorax borkumensis*^46^, was identified with a similar reads abundance profile (PM—0.37%, POW—0.19%, MW—0.59%). Interestingly, this species was shown to degrade both petroleum^47^ and low density polyethylene (LDPE)^48^. These two species were completely or almost completely absent from the open water sample.

### The polyethylene eukaryotic community

**Figure 3:**
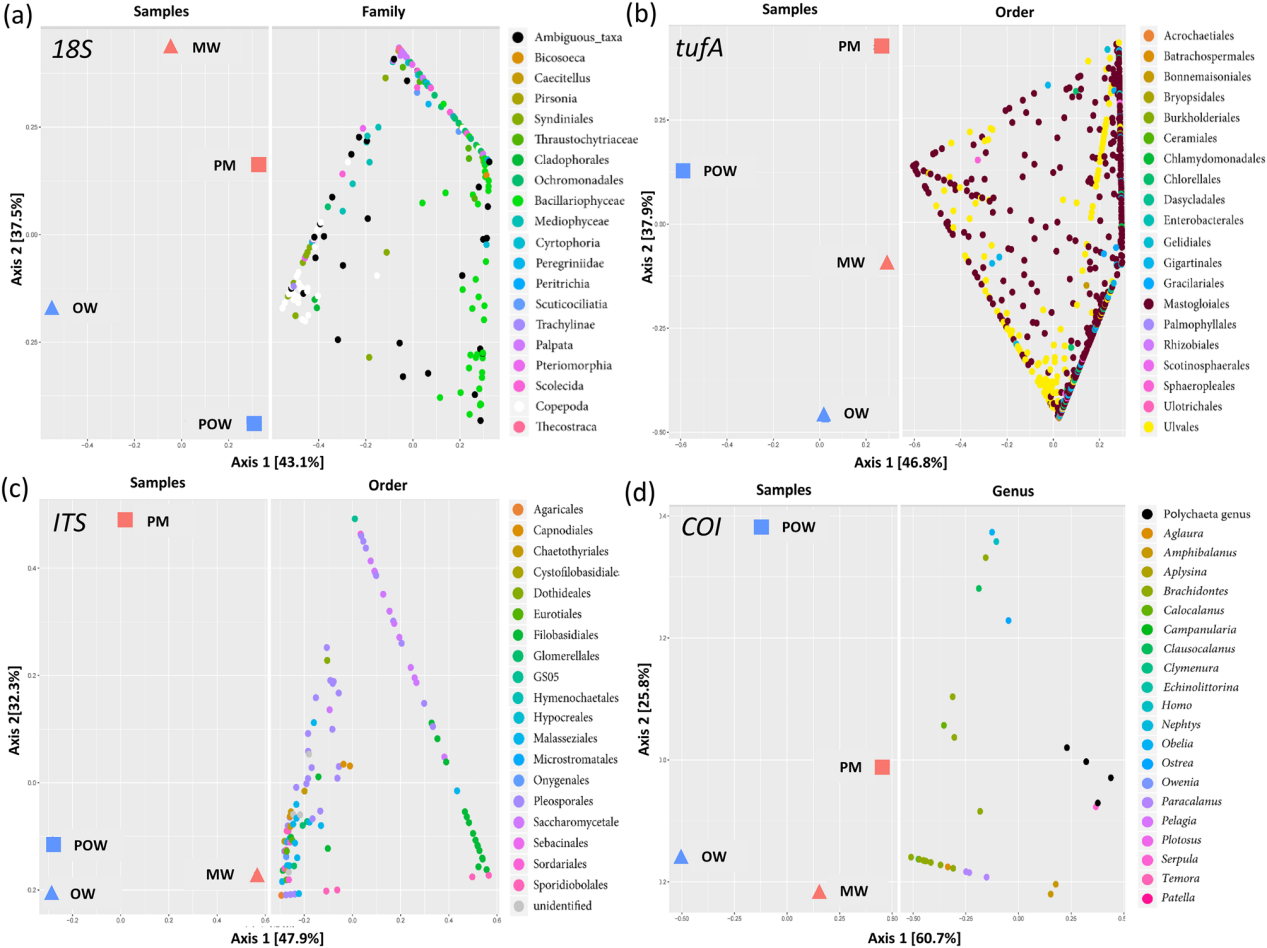
Eukaryote taxa diversity. Beta-diversity of eukaryotic taxa and their distribution among the pooled samples. (**a**) *18S* metabarcoding showing enrichment of diatoms (*Bacillariophyceae*) on PE—in light green. (**b**) *tufA* metabarcoding. (**c**) ITS metabarcoding (**d**) COI metabarcoding. *OW* open water samples, *POW* open water plastic samples, *MW* marina water samples, *PM* marina plastic samples.

**Figure 4:**
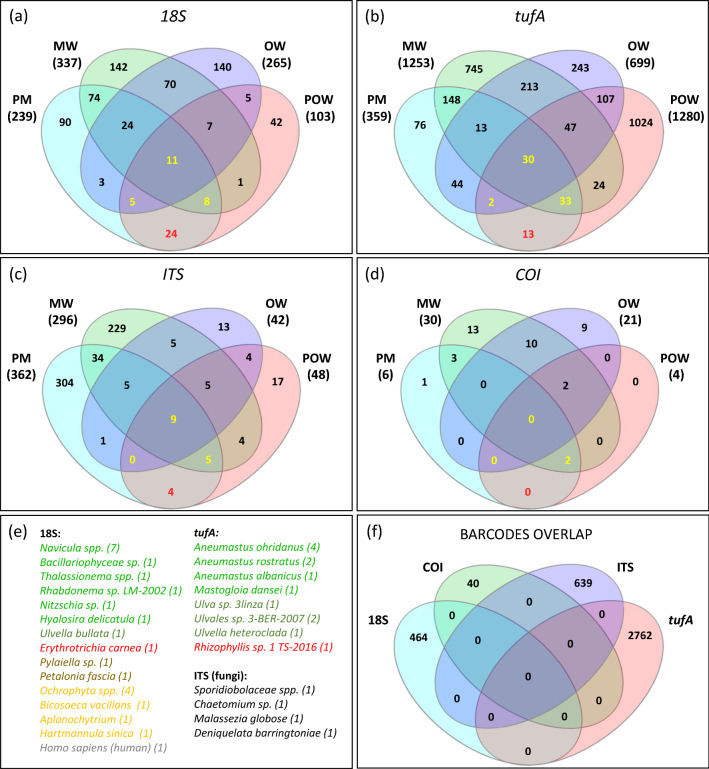
Shared and unique eukaryotic OTUs among the samples. Venn diagrams are presented for each of the four eukaryotic barcodes: (**a**) 18S. (**b**) *tufA*. (**c**) ITS. (**d**) COI. In yellow—OTUs that were identified in both plastic samples, but also in water samples. In red—OTUs that were unique to both plastic samples. *OW* open water samples, *POW* open water plastic samples, *MW* marina water samples, *PM* pooled marina plastic samples, *OTU* counts refer to OTUs that corresponded to ≥ 2 reads. (**e**) List of plastic-specific eukaryote species and the number of their corresponding OTUs (in parentheses). In light green—diatoms, in dark green—green algae, in red—red algae, in brown—brown algae, in yellow—SARs clade protists (f) No shared OTUs were found among the four eukaryote barcodes.

The number of eukaryotic species identified for each of the four sample pools varied between 88 and 280 species for 18S rRNA barcode, 46–186 for ITS barcode, 65–156 for *tufA* barcode and 3–16 for COI barcode. The mapping rates were 4.5–23.3%, 0.5–23.7%, 9.7–43% and 0.03–3.5% accordingly (Table S1). A comparison between the datasets of the four barcodes did not find any taxonomic overlaps (Fig 4f), suggesting that the primers of each barcode target different eukaryote taxa. Overall, more species were identified in the water samples compared to the plastic samples, except fungi (ITS), for which more species were identified in the marina samples, both on PE and free-living (Table S1). The eukaryotic diversity among the samples did not imply a clear taxonomic separation of the PE samples from the water samples and tended to cluster according to the environment they were taken from (marina vs. open water) rather than according to their type (PE-attached vs. free-living) (Fig. 3).

**Figure 5:**
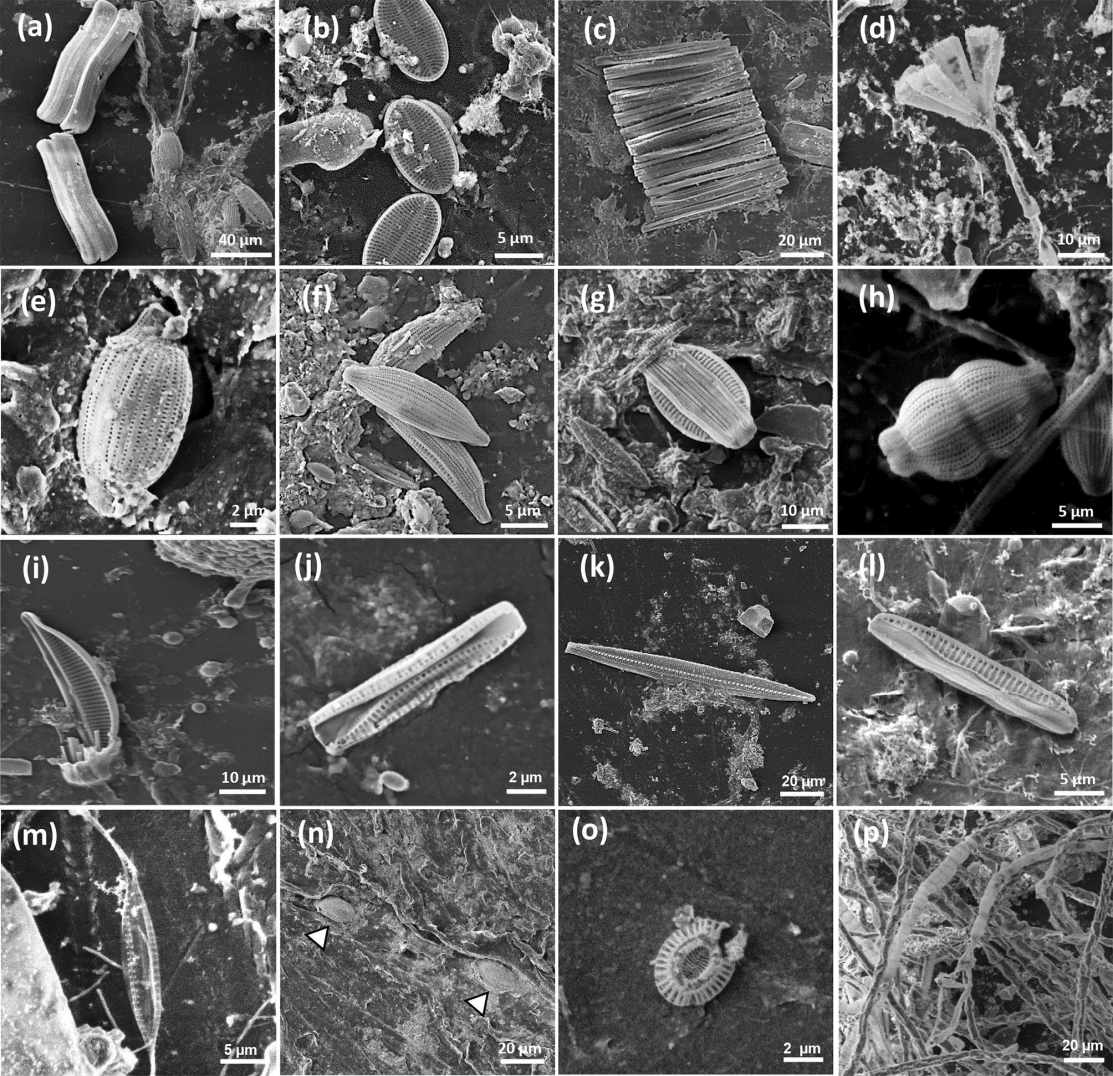
SEM images of common eukaryotic taxa on polyethylene after 1 month. (**a**) *Achnanthes* sp. (**b**) *Nevicula* sp. (**c**) *Fragilaria* sp. (**d**) *Licmophora* sp. (**e**–**i**) *Amphora* sp. (**j**–**l**) *Nitzschia* sp. (**m**) *Cylindrotheca closterium*. (**n**) diatoms dug in the PE surface (white triangles). (**o**)* Coccolithales* sp. (**p**) *Ulvales* sp.

The diatoms were the most dominant eukaryotic microorganisms in the PE samples as identified by the 18S barcoding (Class: *Bacillariophyceae*) (Fig. 3a) and by the *tufA* barcoding (order: *Mastogloiales*) (Fig. 3b) as well as by electron microscopy (Fig. 5). The major PE-associated diatom genera identified were: *Navicula*, *Achnanathes*, *Amphora*, *Nitzschia*, *Rhaphoneis*, *Cylindrotheca* and *Ochrophyta* (18S barcode, Fig. S4) and *Aneumastus* (*tufA* barcode, Fig. S5). In contrast to the diatoms, other taxa, such as the *Copepods*, were restricted to the open water samples and were absent from the PE samples (Fig. 3a). The ITS dataset included fungi from different orders with several PE-enriched species of order *Pleosporales* (Fig. 3C, Fig S6). The COI barcoding resulted in only a few taxa perhaps due to the low mapping rates for this barcode (Table S1). Nevertheless, this analysis identified the *Polychaetas* as an enriched taxon in the marina PE sample (Fig. 3d).

To identify eukaryotic species with a high association to the plastic, we sorted OTUs that were found in the PE samples from both locations but not in water (as was done for bacteria, Fig. 4). The analysis revealed 27 PE-specific species, corresponding to 41 OTUs (Fig. 4, in red), accounting for 0.22% of all eukaryote OTUs in the samples. The plastic-specific taxa included 10 diatom species, 4 green algae, 2 red algae, 2 brown algae, 4 SARs clade protists, 4 fungi as well as human (Fig. 4e). Within these, the dominant species, based on their relative read abundances, were diatoms of *Navicula* (Fig. S4) (Fig. 5) *and Aneumastus* (Fig. S5) genera and fungi of the order *Sporidiobolaceae* (Fig. S6).

## Discussion

Plastic debris in the world oceans is now more abundant than any other type of marine debris, creating a new man-made ecosystem^5^. This ecosystem has been rapidly occupied by rich communities of microorganisms, of which research is still in its infancy. An emerging powerful tool to study the plastisphere biodiversity is DNA metabarcoding^11^. Given the limitations of the available databases, the success and efficiency of DNA metabarcoding are affected by several factors, including the sequencing technology which is being used. In this sense, Nanopore MinION technology has several advantages over the commonly used second-generation sequencing technologies, as suggested both empirically^10,41,49^ and practically^50,51^. Advantages include the high median length of its reads, its portability, its low cost, its simplicity of operation and the fact that it is controllable and provides real-time sequencing data. Although sequencing error rates within single MinION reads are still higher compared to those produced by other platforms, they appear at random positions along the sequence (in contrast to amplification-based NGS techniques), and therefore may be corrected given sufficient coverage^52^. In this study, we successfully tested and implemented a tailored pipeline for the metabarcoding of the plastisphere microbiome with Nanopore MinION sequencing using longer regions of five different barcode loci that allowed a wide coverage of marine prokaryotic and eukaryotic taxa.

Since the purpose of the work was to identify marine microorganisms that preferentially inhabit plastic, we compared the microbiome of pooled PE samples from plastic food bags that were submerged for 1 month at sea to the microbiome of the surrounded water. The plastic bags were positioned at two proximal but environmentally different locations: the marina location, which is protected from high-energy disturbances such as waves and currents, yet exposed to petroleum and other pollutants, and the open water location which is a high energy environment with high a water turnover and lower presence of pollutants. We assumed that only microorganisms with high preference for life on plastic will be found in both of them. Indeed, we identified only 34 bacteria species and 27 eukaryotic species in this category. Among these plastic-specific microorganisms we identified at least 16 bacteria species that are known to be associated with hydrocarbon degradation, mainly of genera *Alcanivorax*, *Acidovorax* and *Alteromonas*^41–43^ and a fungi species, of the order *Sporidiobolaceae* which was previously found to be associated with marine tar^53^. Interestingly, petroleum-associated bacteria taxa, including *Pseudoalteromonas*^44^ and *Alcanivorax*^54^, were abundant in the marina water in addition to their presence in the PE samples. This result fits well with the high abundance of petroleum pollutants in the marina water. Moreover, *A. borkumensis* has recently shown to be able to degrade PE^48^. The presence of this species on plastic and in a petroleum-rich environment at the same time, as suggested by our results, correlates well with its recorded carbohydrate metabolism activity.

While the metabarcoding of eukaryotes is far more challenging than that of the prokaryotes, our results suggest that a combination of several barcodes and different taxonomic databases has the potential to provide a more comprehensive picture of the plastisphere eukaryotic microbiome. In this sense, the *tufA* barcoding complemented the 18S barcoding in identifying the green algae repertoire as well as a few protists that were not probed with the 18S barcode. The ITS barcoding was necessary to cover the fungi Kingdome and the COI barcode, although resulted in low mapping rates, was proven to be of added value in the identification of metazoans.

To complement the eukaryote metabarcoding data we used scanning electron microscopy (SEM) to visualize fixed PE samples from the bags that were used in our experiment. SEM imaging was shown to be useful to understand the composition of the microbiota and the structure of the plastisphere^55–57^. Whereas bacteria species are very hard to be classified based on morphology, certain Protista have certain identifiable morphological characteristics enabling taxonomical classification, at least to a certain resolution. This includes the diatoms (Class *Bacillariophyceae*) that were highly abundant on the plastic surface and can be classified based on the unique structure of their frustule. It is important to note that while in this study we used SEM strictly for the taxonomic identification, this imaging technique has the potential to be very informative for many other aspects of the plastisphere characterization, such as the interactions among plastisphere species, the development and the structure of the biofilm and the changes to the plastic surface itself.

Following our results, we believe that our approach and work pipeline were efficient in the characterization of the plastisphere and the identification of plastic-specific species. Future studies of the plastisphere will benefit from combining multiple molecular and microscopy techniques. Other than metabarcoding, high throughput molecular screening may include metagenomics, transcriptomics, proteomics and metabolomics to characterize the plastisphere composition. Additional visualization techniques, such as fluorescent in situ hybridization (FISH)^58^, may also be applied in plastisphere studies.

## Supplementary information

Supplementary Information.
