# Supplementary material for: Identification of plastic-associated species in the Mediterranean Sea using DNA metabarcoding with Nanopore MinION
Source: Sci Rep. 2020 Oct 16;10:17533. doi: 10.1038/s41598-020-74180-z (PMC7568539; doi:10.1038/s41598-020-74180-z)
Supplement: Supplementary file 1 — Supplementary Information. [file 41598_2020_74180_MOESM1_ESM.docx]

Identification of plastic-associated species in the Mediterranean Sea using metabarcoding with Nanopore MinION – Supporting information

Keren Davidov^1*^, Evgenia Iankelevich-Kounio^1*^, Iryna Yakovenko^1^, Yuri Kochrov^1^, Maxim Rubin^2^, Matan Oren^1**^

**Affiliations:**

^1^ Department of Molecular Biology, Ariel University, Science Park, Ariel 40700, Israel

^2^ Israel Oceanographic and Limnological Research, National Institute of Oceanography, Tel Shikmona, P.O. Box 8030, Haifa 31080, Israel

* equal authorship

** Correspondence to: [matanor@ariel.ac.il](mailto:matanok@ariel.ac.il)

**Supporting information**

**Figure S1**

(a)

(b)

**Figure S1. Read length distribution of barcode amplicons sequenced with MinION.** (a) read length distribution of the 1th MinION multiplexed run. Picks match COI, 18S and 16S amplicon lengths (b) read length distribution of the 2^nd^ multiplexed MinION run. Picks match ITS and *tufA* amplicon lengths.


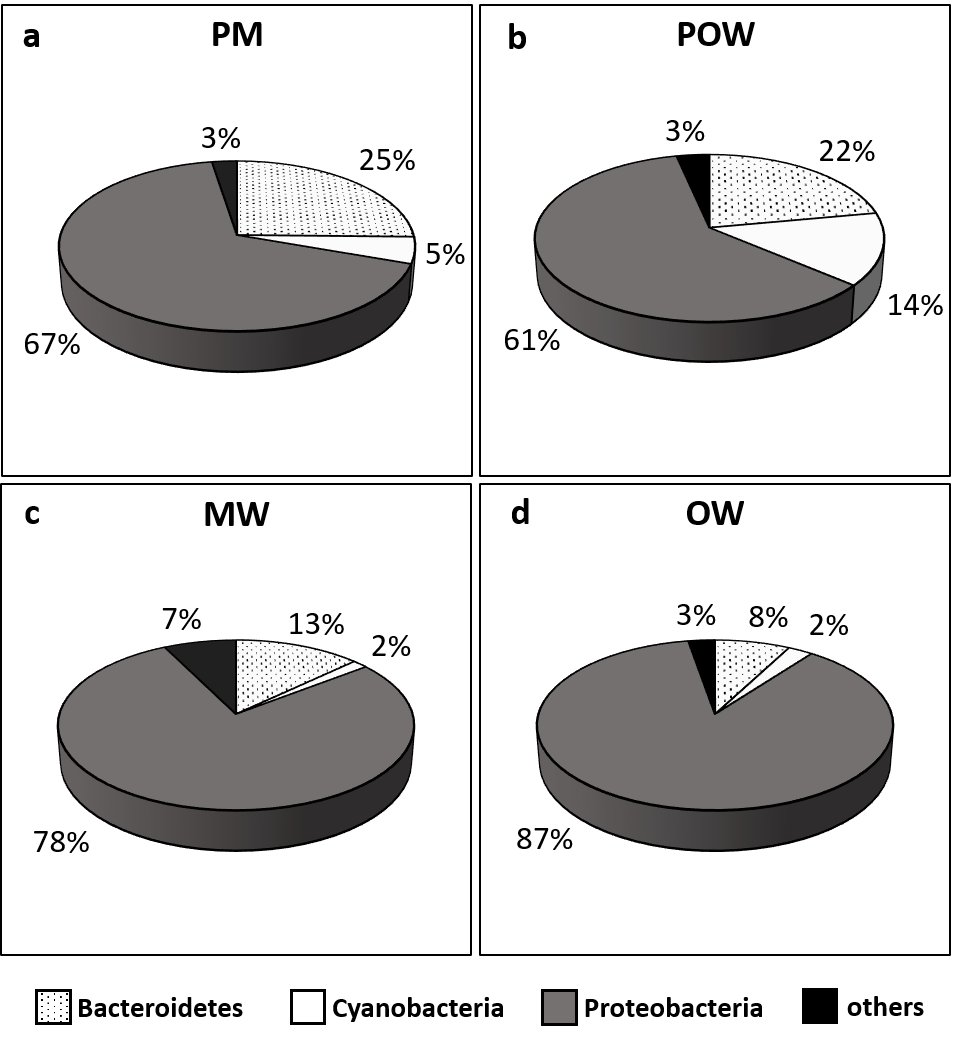
**Figure S2**

**Figure S2. Major bacterial phyla representation in the samples as obtained by metabarcoding**. a and b: polyethylene samples, c and d: water samples. PM. plastic from the marina location. POW. Plastic from the open water location MW. marina water OW. Open water


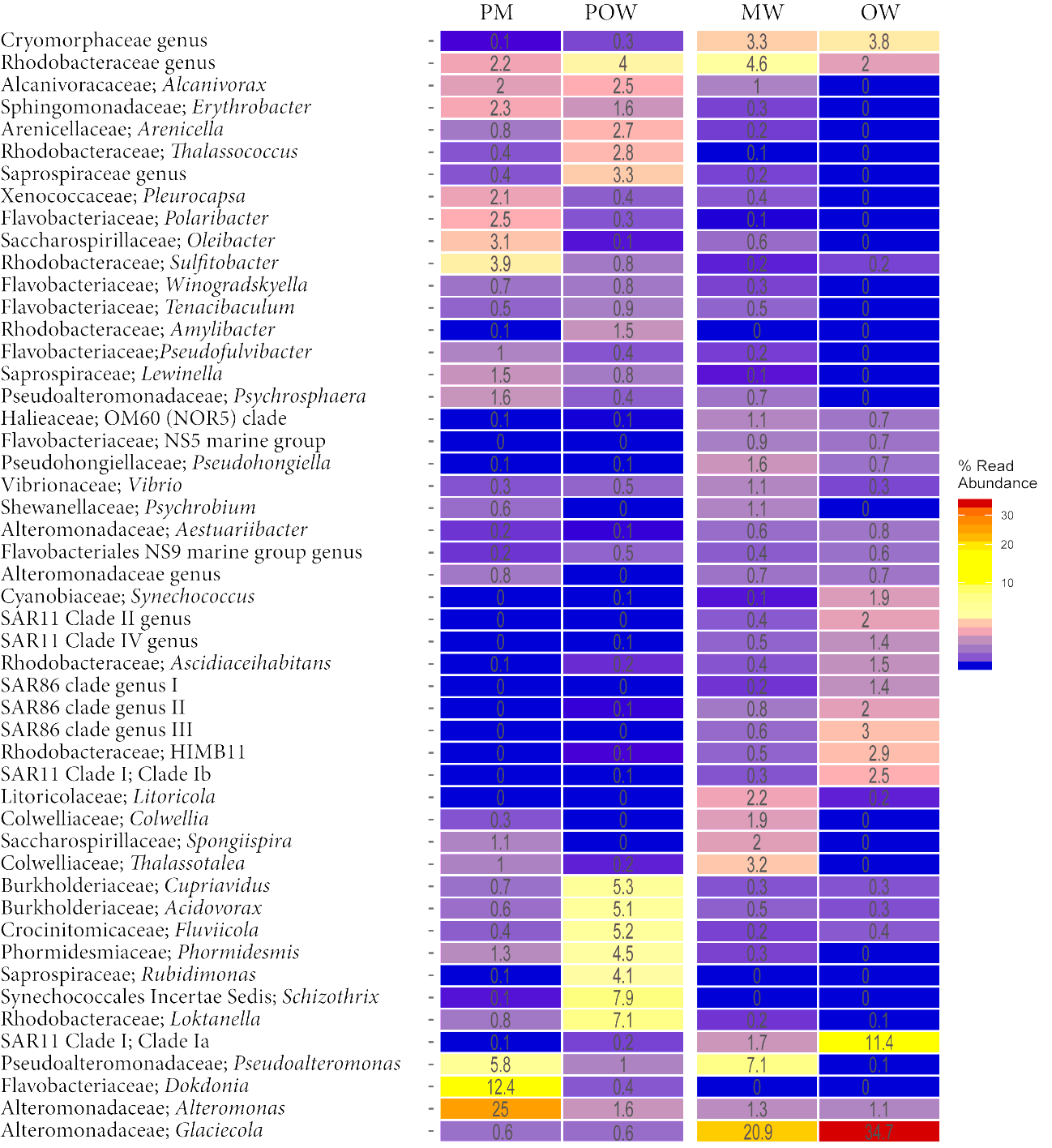


**Figure S3. Heat map of major bacterial taxa in the plastic and water samples.** PM. plastic form marina, POW. plastic from open sea, MW. marina water, OW. open water.


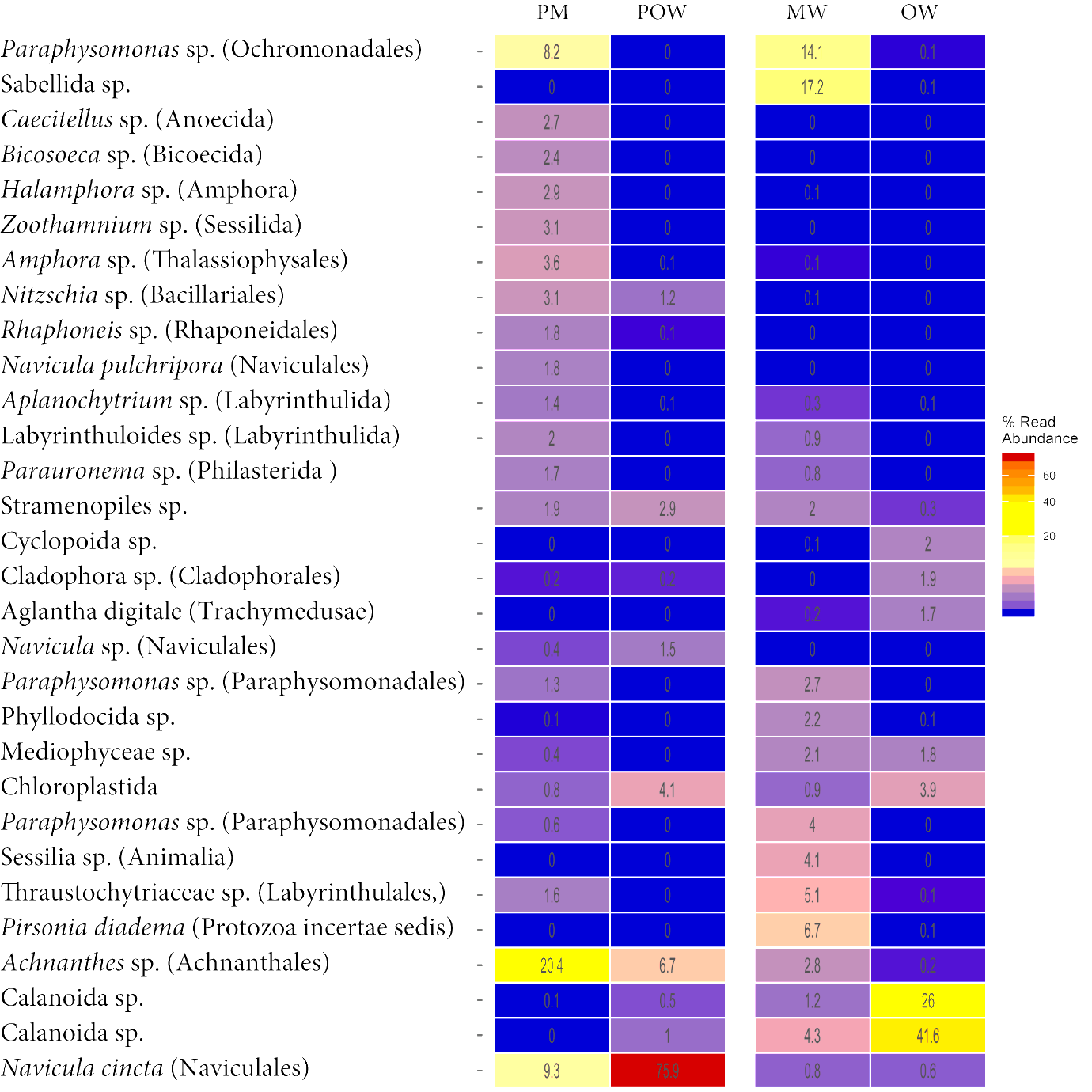


**Figure S4. Heat map of major eukaryote taxa amplified by *18S* from the plastic and water samples.** PM. plastic form marina, POW. plastic from open sea, MW. marina water, OW. open water.


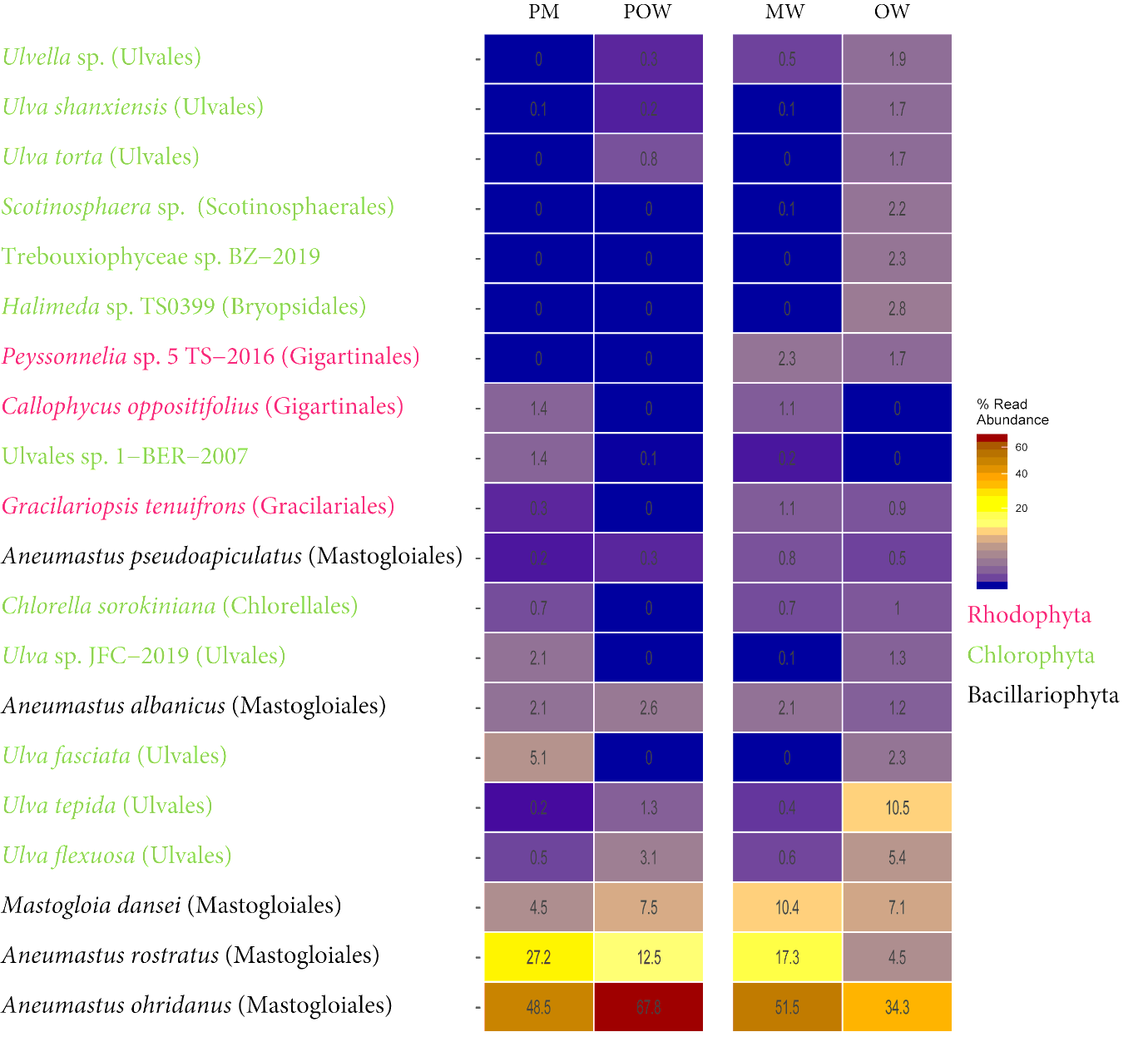


**Figure S5. Heat map of major eukaryote taxa amplified by *tufA* from the plastic and water samples.** PM. plastic form marina, POW. plastic from open sea, MW. marina water, OW. open water.


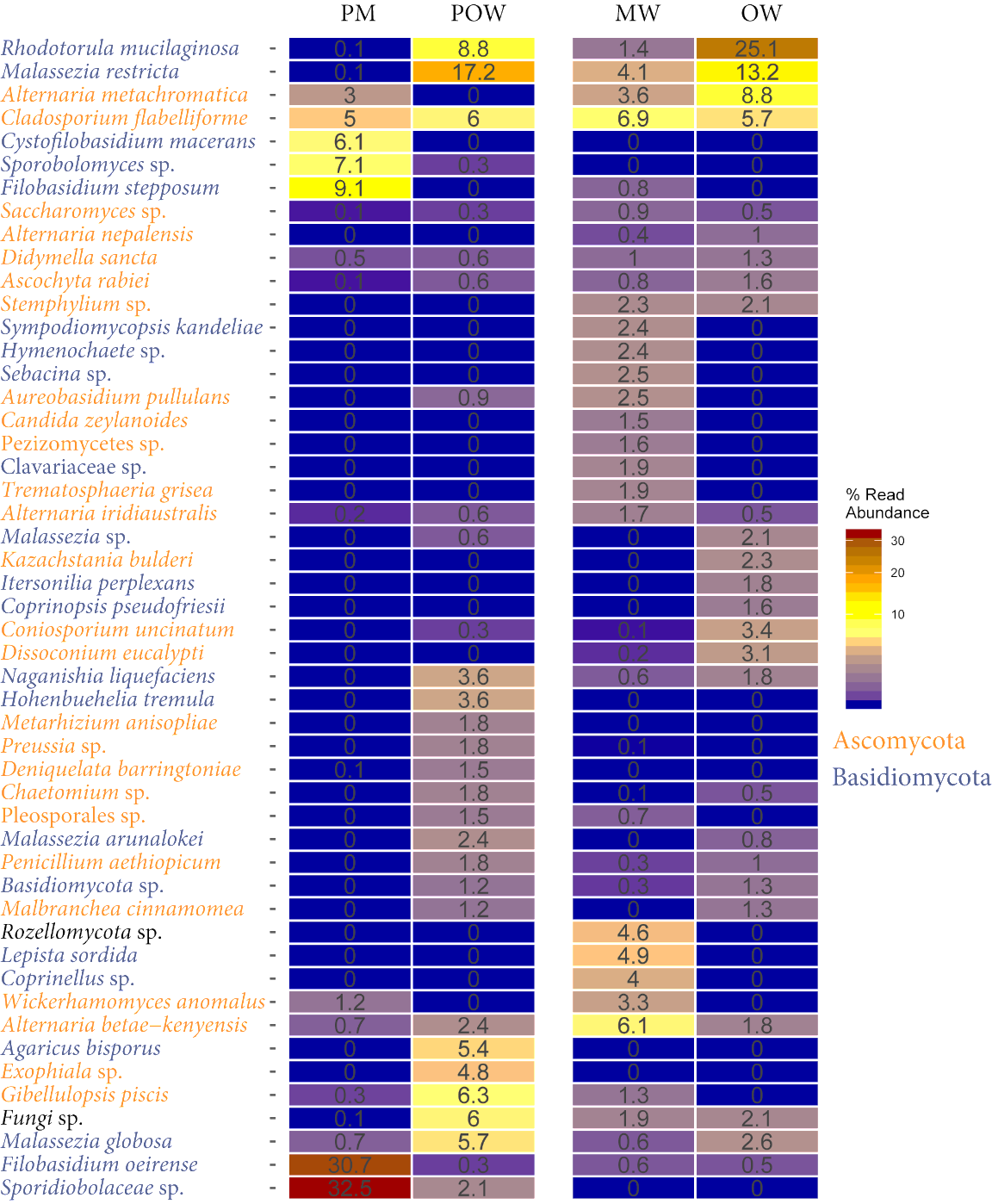


**Figure S6. Heat map of major eukaryote taxa amplified by *ITS* from the plastic and water samples.** PM. plastic form marina, POW. plastic from open sea, MW. marina water, OW. open water.

–
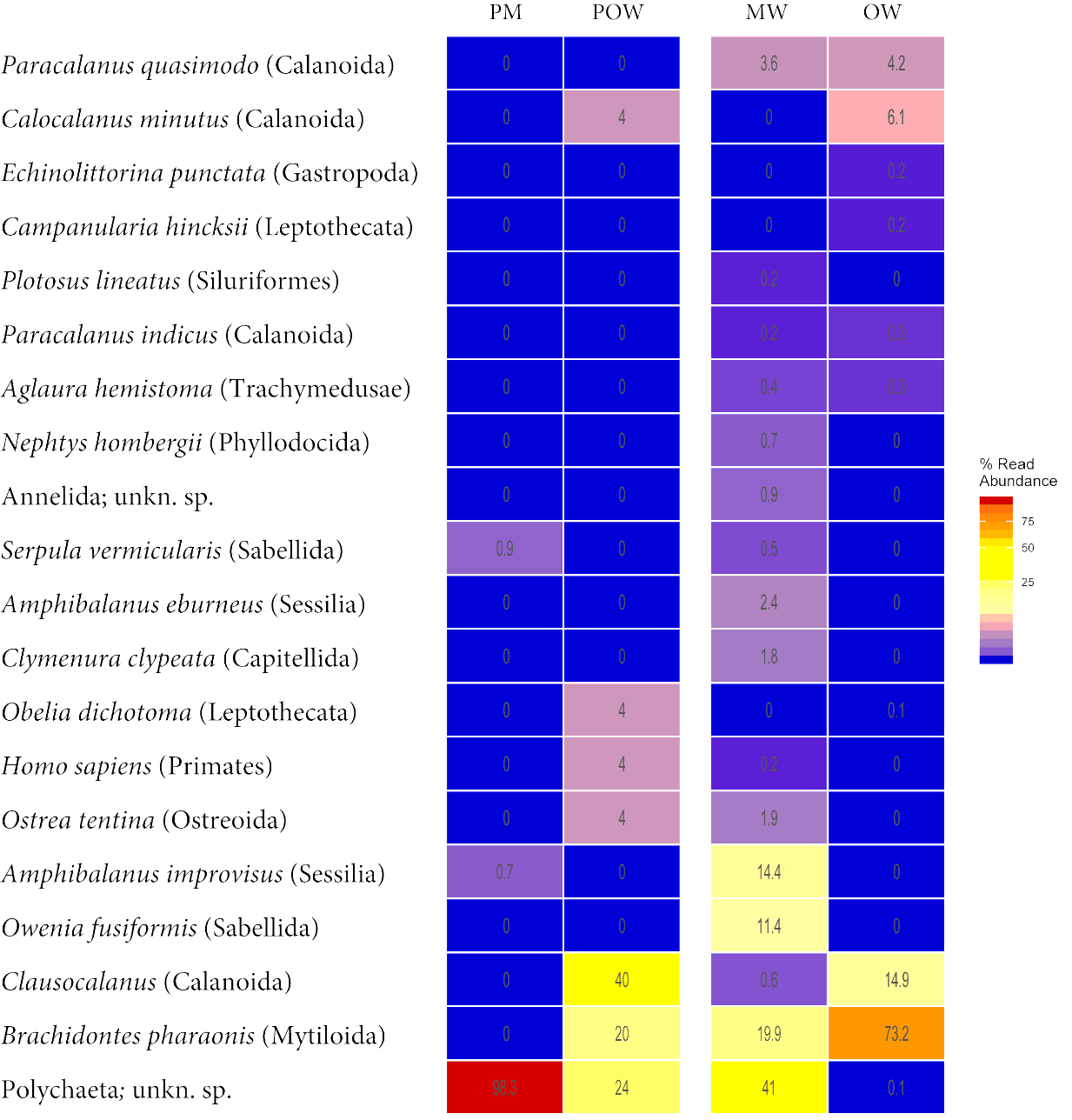


**Figure S7. Heat map of major eukaryote taxa amplified by COI from the plastic and water samples.** PM. plastic form marina, POW. plastic from open sea, MW. marina water, OW. open water.

**Supplementary tables**

**Table S1. General run parameters**

| No' of species | No' of OTUs* | Mapped reads | Total reads | Marker | Sample |
| --- | --- | --- | --- | --- | --- |
| 1,823 | 10,773 | 26,981 | 42,324 | 16s | **MW** |
| 280 | 522 | 12,108 | 51,924 | 18s |  |
| 16 | 40 | 1,076 | 30,664 | COI |  |
| 186 | 878 | 3,857 | 69,328 | ITS |  |
| 156 | 3,799 | 38,689 | 217,953 | tufA |  |
| 2,572 | 137,529 | 192,533 | 315,399 | 16s | **OW** |
| 183 | 455 | 7,730 | 173,129 | 18s |  |
| 16 | 35 | 1,153 | 85,350 | COI |  |
| 53 | 142 | 387 | 74,603 | ITS |  |
| 135 | 2,224 | 15,139 | 156,549 | tufA |  |
| 1,810 | 41,312 | 56,501 | 111,917 | 16s | **PM** |
| 190 | 357 | 6,233 | 111,708 | 18s |  |
| 3 | 8 | 537 | 70,661 | COI |  |
| 73 | 1,658 | 14,376 | 60,540 | ITS |  |
| 65 | 1,178 | 11,726 | 31,010 | tufA |  |
| 1,004 | 8,334 | 10,363 | 244,021 | 16s | **POW** |
| 88 | 157 | 9,938 | 98,645 | 18s |  |
| 7 | 11 | 25 | 68,701 | COI |  |
| 46 | 120 | 331 | 64,169 | ITS |  |
| 104 | 6,818 | 67,002 | 155,751 | tufA |  |

* Total OTU counts for each genetic barcode in each of the pooled samples. The numbers are different from the numbers presented in the Venn diagrams in figures 2 and 4, where only OTUs corresponding to ≥ 2 reads were included.

**Table S2. PE-enriched prokaryote taxa and their relative abundancies**

| Taxa | PM (%) | POW (%) | MW (%) | OW (%) |
| --- | --- | --- | --- | --- |
| *Pseudoalteromonas sp. 12* | 0.554 | 0.068 | 1.950 | 0.004 |
| *Cupriavidus sp.* | 0.230 | 1.785 | 0.230 | 0.081 |
| *Dokdonia sp. PRO95* | 1.349 | 0.019 | 0.007 | - |
| *Alcanivorax borkumensis* | 0.372 | 0.193 | 0.593 | - |
| *uncultured Fluviicola sp.* | 0.080 | 1.042 | 0.026 | 0.008 |
| *uncultured Arenicella sp.* | 0.179 | 0.772 | 0.096 | - |
| *uncultured Acidovorax sp.* | 0.104 | 0.724 | 0.104 | 0.031 |
| *uncultured Pseudoalteromonas sp.* | 0.189 | 0.048 | 0.582 | 0.005 |
| *uncultured Ascidiaceihabitans sp.* | 0.027 | 0.116 | 0.196 | 0.247 |
| *gamma proteobacterium 14III/A01/016* | 0.216 | 0.068 | 0.208 | - |
| *Alcanivorax sp. OM-2* | 0.097 | 0.299 | 0.026 | - |
| *uncultured Flavobacteriales bacterium* | 0.071 | 0.039 | 0.159 | 0.139 |
| *Thalassococcus halodurans* | 0.055 | 0.338 | - | - |
| *Pseudoalteromonas sp. JL1130* | 0.025 | 0.019 | 0.319 | 0.003 |
| *Ruegeria sp. 3X/A02/236* | 0.009 | 0.318 | 0.007 | - |
| *Acidovorax valerianellae* | 0.037 | 0.232 | 0.041 | 0.011 |
| *Pseudoalteromonas sp. 36* | 0.069 | 0.019 | 0.230 | - |
| *Acidovorax sp. R-24667* | 0.016 | 0.203 | 0.056 | 0.007 |
| *uncultured Altererythrobacter sp.* | 0.021 | 0.212 | 0.044 | - |
| *uncultured Arenicellaceae bacterium* | 0.060 | 0.164 | 0.041 | - |
| *uncultured Woeseia sp.* | 0.007 | 0.029 | 0.219 | 0.005 |
| *uncultured Vibrio sp.* | 0.007 | 0.039 | 0.208 | 0.005 |
| *uncultured Marinicella sp.* | 0.087 | 0.048 | 0.115 | - |
| *Tenacibaculum sp. MOLA 533* | 0.058 | 0.048 | 0.137 | 0.003 |
| *uncultured Saprospiraceae bacterium* | 0.028 | 0.193 | 0.022 | - |
| *Tenacibaculum aestuarii* | 0.030 | 0.116 | 0.096 | 0.001 |
| *uncultured Rubidimonas sp.* | 0.004 | 0.222 | 0.007 | - |
| *Loktanella sp. S4079* | 0.007 | 0.203 | 0.007 | - |
| *Erythrobacter nanhaisediminis* | 0.071 | 0.096 | 0.044 | - |
| *uncultured Erythrobacter sp.* | 0.065 | 0.096 | 0.026 | - |
| *uncultured Hyphomonadaceae bacterium* | 0.007 | 0.164 | - | - |
| *uncultured Ruegeria sp.* | 0.005 | 0.039 | 0.126 | 0.001 |
| *uncultured Polaribacter 4 sp.* | 0.030 | 0.096 | 0.007 | - |
| *Phormidesmis priestleyi] ANT.LPR2.6* | 0.005 | 0.106 | 0.022 | - |
| *Hyphomonas polymorpha PS728* | 0.012 | 0.087 | 0.011 | - |
| *uncultured Aliiglaciecola sp.* | 0.014 | 0.019 | 0.059 | - |
| *Stanieria sp. UE7A* | 0.019 | 0.019 | 0.052 | - |
| *Alteromonas mediterranea MED64* | 0.030 | 0.048 | - | - |
| *Alcanivorax sp. Haw1* | 0.027 | 0.048 | - | - |
| *uncultured Nisaea sp.* | 0.004 | 0.068 | - | - |
| *Roseobacter sp. CCS2* | 0.004 | 0.058 | 0.007 | - |
| *Alteromonas sp.* | 0.028 | 0.029 | - | - |
| *uncultured Pseudofulvibacter sp.* | 0.027 | 0.029 | - | - |
| *uncultured Olleya sp.* | 0.007 | 0.048 | - | - |
| *uncultured Phormidesmiaceae bacterium* | 0.011 | 0.019 | 0.022 | - |
| *uncultured Lewinella sp.* | 0.004 | 0.048 | - | - |
| *uncultured Alteromonas sp.* | 0.011 | 0.039 | - | - |
| *uncultured Crocinitomix sp.* | 0.005 | 0.029 | 0.007 | - |
| *uncultured Rhodobacteraceae bacterium* | 0.011 | 0.029 | - | - |
| *uncultured planctomycete* | 0.005 | 0.019 | 0.011 | 0.004 |
| *Erythrobacter sp. SY-46* | 0.012 | 0.019 | 0.007 | - |
| *uncultured Winogradskyella sp.* | 0.009 | 0.029 | - | - |
| *uncultured Streptococcus sp.* | 0.005 | 0.019 | 0.011 | 0.002 |
| *Alteromonas sp. NJSX51* | 0.007 | 0.029 | - | - |
| *Acidovorax sp. B4* | 0.004 | 0.029 | - | - |
| *Marinovum sp. YP194* | 0.004 | 0.029 | - | - |
| *Tenacibaculum sp. HJ103* | 0.004 | 0.029 | - | - |
| *uncultured Litorimicrobium sp.* | 0.005 | 0.019 | 0.007 | - |
| *marine bacterium Tw-3* | 0.004 | 0.019 | 0.007 | - |
| *Arenicella chitinivorans* | 0.005 | 0.019 | - | - |
| *Loktanella litorea* | 0.005 | 0.019 | - | - |
| *uncultured Loktanella sp.* | 0.005 | 0.019 | - | - |
| *Acidovorax sp.* | 0.004 | 0.019 | - | - |
| *Kordia algicida OT-1* | 0.004 | 0.019 | - | - |
| *Octadecabacter sp. CAU 1310* | 0.004 | 0.019 | - | - |
| *Roseobacter sp.* | 0.004 | 0.019 | - | - |
| *uncultured Litorimonas sp.* | 0.004 | 0.019 | - | - |
